# Comparison of Postoperative Analgesic Efficacy Between Opioid-free Anaesthesia Using Intravenous Magnesium, Lidocaine, and Erector Spinae Plane Block, and Opioid-based Anaesthesia in Laparoscopic Abdominal Surgeries: A Randomised Controlled Trial

**DOI:** 10.4274/TJAR.2026.241747

**Published:** 2026-10-30

**Authors:** Jerfiha VJ, Ezhilrajan Vaithiyalingam, Pradeesh Johny

**Affiliations:** 1 Aarupadai Veedu Medical College & Hospital Clinic of Anaesthesiology, Puducherry, India

**Keywords:** Fentanyl, lidocaine, magnesium, opioid, pain, regional anaesthesia

## Abstract

**Objective:**

Opioid-based anaesthesia is a standard practice in laparoscopic surgeries. However, it is associated with adverse effects that can limit its use. To address these issues, opioid-free anaesthesia has been introduced as an alternative. This study aims to compare the analgesic efficacy of opioid-free anaesthesia, incorporating magnesium, lidocaine, and an erector spinae plane block (ESPB), with traditional opioid-based anaesthesia during laparoscopic procedures.

**Methods:**

Seventy patients aged 18-60 years undergoing laparoscopic surgery were randomly divided into two groups of 35 each. Opioid-free group received opioid-free anaesthesia, which included an intravenous (IV) bolus of 2 g magnesium, a continuous lidocaine infusion at 1.5 mg kg^-1^ hr^-1^, and a bilateral ESPB with 25 mL of 0.25% bupivacaine. Opioid-based group received opioid-based anaesthesia with an IV dose of 2 µg kg^-1^ fentanyl. Postoperative pain and the need for rescue analgesia were assessed for 24 h. Perioperative haemodynamic parameters were recorded, and the incidence of postoperative complications, including sedation, nausea, vomiting, and shivering, was monitored for 24 h.

**Results:**

Pain scores were significantly lower in the opioid-free group compared to the opioid-based group at 0 h (*P* < 0.001), 2 h (*P* < 0.001), and 4 h (*P*=0.005) postoperatively. The opioid-based group required rescue analgesia more frequently (*P* < 0.001). Additionally, the incidence of postoperative complications was significantly lower in the opioid-free group than in the opioid-based group (*P* < 0.001). Haemodynamic parameters were similar between the two groups.

**Conclusion:**

Opioid-free anaesthesia provides superior postoperative analgesia and reduces the incidence of postoperative complications compared with opioid-based anaesthesia for laparoscopic surgery.

**Main Points**

• Opioid-free anaesthesia provides better postoperative analgesia than opioid-based anaesthesia.

• The requirement for rescue analgesia is lower in the opioid-free group than in the opioid-based group.

• The incidence of postoperative complications was lower in the opioid-free group.

## Introduction

Laparoscopic procedures are widely preferred for their minimally invasive approach, which leads to reduced postoperative pain and faster recovery compared to open surgery. However, the early postoperative period may still involve pain owing to tissue injury. Peritoneal irritation from pneumoperitoneum and residual blood can also cause considerable discomfort.^1^ These surgeries are typically performed under general anaesthesia, with opioids used to blunt the intubation response and manage perioperative pain. Despite their sympatholytic benefits, opioids are associated with adverse effects such as sedation, respiratory depression, vomiting, dependence, and tolerance.^2^ To address these concerns, opioid-free anaesthesia has recently become a popular approach to perioperative pain management.^3^ The erector spinae plane block (ESPB) achieves analgesia by blocking the ventral and dorsal rami of thoracic spinal nerves.^4^ Lidocaine and magnesium are also used for managing perioperative pain. We hypothesised that opioid-free anaesthesia, incorporating magnesium, lidocaine, and ESPB, would provide superior postoperative analgesia compared to opioid-based anaesthesia. The primary objective of this study was to assess the time to first postoperative rescue analgesia as a measure of analgesic efficacy. Secondary objectives included monitoring perioperative hemodynamic parameters and postoperative complications such as sedation, nausea, vomiting, and shivering.

Current literature on the use of the ESPB as part of opioid-free anaesthesia in laparoscopic surgeries is limited. This study aims to promote the broader adoption of ESPB in these procedures. In this context, we conducted the study to evaluate the analgesic efficacy of opioid-free anaesthesia, including intravenous (IV) magnesium, lidocaine, and ESPB, compared with opioid-based anaesthesia with fentanyl during laparoscopic surgery.

## Methods

This prospective, randomised, controlled trial was performed at a tertiary care hospital and enrolled 70 patients aged 18-60 years with American Society of Anesthesiologists (ASA) physical status I-II who were undergoing laparoscopic cholecystectomy or appendectomy. The study commenced after receiving approval from the Aarupadai Veedu Medical College & Hospital Institutional Human Ethical Committee (approval no: AV/IHEC/2022/061, date: 18.08.2022) and after being registered with the Clinical Trial Registry of India (CTRI/2022/10/046738).

The study period spanned from November 2022 to May 2024. Patients were excluded if they had coagulation abnormalities, infections at the regional block site, hypersensitivity to the administered drugs, a body mass index >35 kg m^-2^, or if they were undergoing emergency surgeries.

After obtaining informed written consent, preoperative assessments were performed and baseline vital signs were recorded for all 70 patients. Patients were randomly allocated to two groups (n = 35 each) using a computer-generated randomisation sequence created by an independent statistician, and group allocation was concealed using sealed opaque envelopes. The opioid-free group received IV lidocaine and magnesium, whereas the opioid-based group received IV lidocaine and fentanyl.

Alprazolam 0.25 mg and pantoprazole 40 mg were administered orally as premedication the night before surgery. Standard ASA fasting guidelines were adhered to before the procedure. In the preoperative area, an IV cannula was inserted, and IV fluids were started. Upon arrival in the operating room, standard ASA monitors were attached, including electrocardiogram, pulse oximetry, and non-invasive blood pressure monitoring. The patients were pre-oxygenated with 100% oxygen for 3 min. Premedication was administered to both groups with IV midazolam 0.05 mg kg^-1^, glycopyrrolate 0.01 mg kg^-1^, and ondansetron 0.1 mg kg^-1^.

Opioid-free group patients received an IV bolus of lidocaine 1.5 mg kg^-1^ and magnesium 2 g before induction. Opioid-based group patients received an IV bolus of lidocaine 1.5 mg kg^-1^ and fentanyl 2 µg kg^-1^ before induction.

In both groups, patients were induced with propofol 2 mg kg^-1^ IV, and succinylcholine 2 mg kg^-1^ IV was administered to facilitate intubation. After intubation, a loading dose of cisatracurium 0.2 mg kg^-1^ IV was given to maintain muscle relaxation. In the opioid-free group, a lidocaine infusion at a rate of 1.5 mg kg^-1^ hr^-1^ was started after intubation, and an ESPB was performed at the T6 level bilaterally with 25 mL of 0.25% bupivacaine while the patient was positioned laterally. Anaesthesia was maintained with a mixture of 50% nitrous oxide, 50% oxygen, and 1% isoflurane to achieve a mean alveolar concentration of 1. Cisatracurium 0.03 mg kg^-1^ IV was administered every 30 min in both groups. In both groups, intraoperative haemodynamic parameters, including heart rate (HR), systolic blood pressure (SBP), diastolic blood pressure (DBP), and mean arterial pressure (MAP), were monitored and recorded at eight time points: baseline, after intubation, before pneumoperitoneum, and at 10, 30, and 60 min after pneumoperitoneum, as well as after carbon dioxide (CO_2_) deflation and after extubation. If HR or MAP increased by more than 20% from baseline, the opioid-free group received an Esmolol 10 mg IV bolus, while the opioid-based group was administered fentanyl 0.5 µg kg^-1^. In the opioid-free group, the lidocaine infusion was discontinued at the beginning of skin suturing. Both groups received neostigmine 0.05 mg kg^-1^ IV and glycopyrrolate 0.01 mg kg^-1^ IV for reversal of neuromuscular blockade. After extubation, patients were transferred to the recovery room for observation and were provided with oxygen support via a face mask. Upon arrival in the recovery room, pain and sedation level were assessed using the Visual Analogue Scale (VAS) and the Ramsay Sedation Scale, respectively. Assessments were then repeated at 2, 4, 6, 12, and 24 h postoperatively. Patients with a VAS score of 5 or higher received 1 g paracetamol IV as rescue analgesia. Postoperative complications such as nausea, vomiting, and shivering were monitored and treated as needed for 24 h.

### Statistical Analysis

The sample size was calculated a priori for the primary outcome (postoperative VAS score) based on data from a previously published study by Ragupathy et al.,^5^ which evaluated postoperative pain using the VAS in patients undergoing laparoscopic surgeries. A clinically meaningful mean difference of 2 VAS units and a standard deviation of 2.3 VAS units were assumed. With a two-sided significance level (α) of 0.05 and a power (1-β) of 80%, the minimum required sample size was 66 participants (33 per group). To account for potential dropouts (anticipated 6-10%), the sample size was increased and rounded up to 35 participants per group, resulting in a total of 70 participants.

Statistical analyses were performed using the Statistical Package for the Social Sciences, version 23.0 (IBM Corp., Armonk, NY, USA). Continuous data were expressed as mean ± standard deviation and compared using the independent samples t-test. Categorical data were presented as counts (percentages) and compared using the chi-square test or Fisher’s exact test, as appropriate. All tests were two-sided, and a *P* value <0.05 was considered statistically significant.

## Results

A total of 83 patients were evaluated for the study, with 13 excluded for not meeting eligibility criteria. The remaining 70 patients were randomly assigned to two groups of 35 each. All participants completed the study (Figure 1). Demographic and patient characteristics were similar across groups (Table 1).

Figure 2 shows the distribution of VAS scores at various postoperative time intervals for each group. VAS scores were significantly higher in the opioid-based group at 0 h (*P* < 0.001), 2 h (*P* < 0.001), and 4 h (*P*=0.005).

The requirement for rescue analgesia was significantly higher in the opioid-based group than in the opioid-free group (*P* < 0.001) (Table 2). However, time to first rescue analgesia did not differ significantly between groups (*P*=0.401) (Table 2).

Mean HR values at different time points were comparable between the two groups, with no statistically significant differences (all *P* > 0.05) (Table 3).

Similarly, baseline SBP, DBP, and MAP were comparable between the groups, with no significant differences observed after intubation or before CO_2_ insufflation (Table 4). However, at 10 and 30 minutes after CO_2_ insufflation, SBP, DBP, and MAP were significantly higher in the opioid-based group compared to the opioid-free group (all *P* < 0.05). No statistically significant differences were observed at 60 minutes after CO_2_ insufflation, after CO_2_ deflation, or after extubation (all *P* > 0.05) (Table 4).

This study found that sedation levels were significantly higher in the opioid-based group compared to the opioid-free group at 2 h (*P*=0.018) and 4 h (*P*=0.031) postoperatively (Figure 3).

Postoperative nausea (*P*=0.074), vomiting (*P*=0.555), and shivering (*P*=0.314) did not differ significantly between the two groups. However, the overall incidence of complications was significantly higher in the opioid-based group than in the opioid-free group (*P*=0.021) (Figure 4).

## Discussion

In this study, we compared the analgesic efficacy of opioid-free anaesthesia with that of opioid-based anaesthesia during laparoscopic surgeries. Our findings showed that patients in the opioid-free group reported lower pain scores than those in the opioid-based group.

Laparoscopic procedures are preferred for their advantages, such as shorter hospital stays and fewer complications; however, managing immediate postoperative pain remains challenging. Pain after laparoscopic procedures can be substantial for up to 6 h postoperatively.^1^ Traditionally, opioids have been used for pain management during these surgeries despite their associated side effects. In response, opioid-free anaesthesia has emerged as an effective alternative that employs a combination of medications, peripheral nerve blocks, and neuraxial analgesia to achieve comparable pain relief and reduce opioid-related complications.^6^

Lidocaine offers several benefits in the perioperative setting, including suppression of the intubation response, reduction of pain, reduction in postoperative nausea and vomiting, and shortening of the duration of ileus.^7, 8^ Its analgesic effects are multifaceted, involving the inhibition of sodium, potassium, and calcium channels, Gαq-coupled protein receptors, N-methyl-D-aspartic acid (NMDA) receptors, and 5-hydroxytryptamine 3 receptors.^9^ Magnesium is also used perioperatively to manage pain, acting through both competitive and non-competitive antagonism of NMDA receptors at hippocampal presynaptic calcium channels, thus preventing central sensitisation.^10^ In the ESPB, local anaesthetics are administered into the deep fascia of the erector spinae muscle at the level of the transverse process to block the dorsal rami of the spinal nerves. Additionally, these anaesthetics diffuse into the paravertebral space, providing further pain relief.^4, 11^

This study found that the opioid-based group had higher VAS scores at 2 and 4 h postoperatively compared to the opioid-free group. Consequently, the opioid-based group required rescue analgesia more frequently during the first 6 h postoperatively. The lower VAS scores in the opioid-free group can be attributed to the extended analgesic effects of the ESPB and to the administration of lidocaine and magnesium.^12^ This finding is consistent with a meta-analysis by Koo et al.,^13^ which reviewed eight randomised controlled trials assessing the postoperative analgesic efficacy of the ESPB in patients undergoing laparoscopic cholecystectomy. The analysis concluded that the ESPB effectively reduces pain scores for up to 12 h postoperatively in this patient group.^13^

Aronsohn et al.^14^ assessed the analgesic efficacy of opioid-free versus opioid-based anaesthesia, with the opioid-free group receiving dexmedetomidine, ketamine, and lignocaine. They found that median pain scores were similar during both the initial 6 h post-surgery and the subsequent 6-24 h.^14^ Based on these findings, we conclude that the extended postoperative pain relief observed in the opioid-free group is mainly attributable to the ESPB rather than to lignocaine and magnesium.

The mean SBP, DBP, and MAP were similar from baseline until CO_2_ inflation. However, these values were considerably higher in the opioid-based group at both 10 and 30 min after pneumoperitoneum. This disparity may be attributed to the superior analgesic efficacy and sympatholytic effects of the ESPB, lidocaine, and magnesium used in the opioid-free group, compared with those of fentanyl. This observation is consistent with the study by Elshafie et al.,^12^ which compared opioid-free anaesthesia involving the ESPB, dexmedetomidine, magnesium, and lidocaine, with opioid-based anaesthesia during hepatic resection. They found that the block group had significantly lower intraoperative blood pressure than the opioid-based group.^12^

The postoperative Ramsay sedation score was higher in the opioid-based group at both 2 and 4 h than in the opioid-free group, likely owing to the pronounced sedative effects of fentanyl. This result contrasts with a study by Choi et al.,^15^ which evaluated recovery quality between opioid-free (dexmedetomidine and lidocaine) and opioid-based (remifentanil) anaesthesia following gynaecological laparoscopy. They found no significant difference in sedation levels between the groups.^15^ Another study by Massoth et al.,^16^ which compared opioid-free and opioid-based anaesthesia after gynaecological surgery, reported higher sedation levels in the opioid-free group, which received dexmedetomidine and esketamine compared with the group receiving sufentanil. The variation in findings may be attributed to the shorter duration of action of remifentanil and sufentanil.

The incidence of postoperative nausea, vomiting, and shivering was similar between the two groups. However, when individual complications were combined, the opioid-based group showed a significantly higher overall incidence of complications than the opioid-free group. This indicates that while the frequencies of individual complications did not differ considerably, the opioid-based group experienced a higher total number of complications. This finding is consistent with the study by Toleska and Dimitrovski^17^ which compared opioid-free and opioid-based anaesthesia during laparoscopic cholecystectomy and found that the opioid-based group had a higher rate of postoperative complications.^17^ However, the small sample size and the focus on only two types of laparoscopic surgery limited the generalizability of these results to other laparoscopic procedures.

## Conclusion

Opioid-free anaesthesia provides superior perioperative analgesia and reduces the incidence of postoperative complications compared to opioid-based anaesthesia in laparoscopic surgeries. We recommend using the ESPB in combination with lidocaine and magnesium to provide effective analgesia during these procedures.

## Ethics

**Ethics Committee Approval:** Approval from the Aarupadai Veedu Medical College & Hospital Institutional Human Ethical Committee (approval no: AV/IHEC/2022/061, date: 18.08.2022) and after being registered with the Clinical Trial Registry of India (CTRI/2022/10/046738).

**Informed Consent:** Written informed consent was obtained from all participants included in the study.

## Figures and Tables

**Figure 1:**
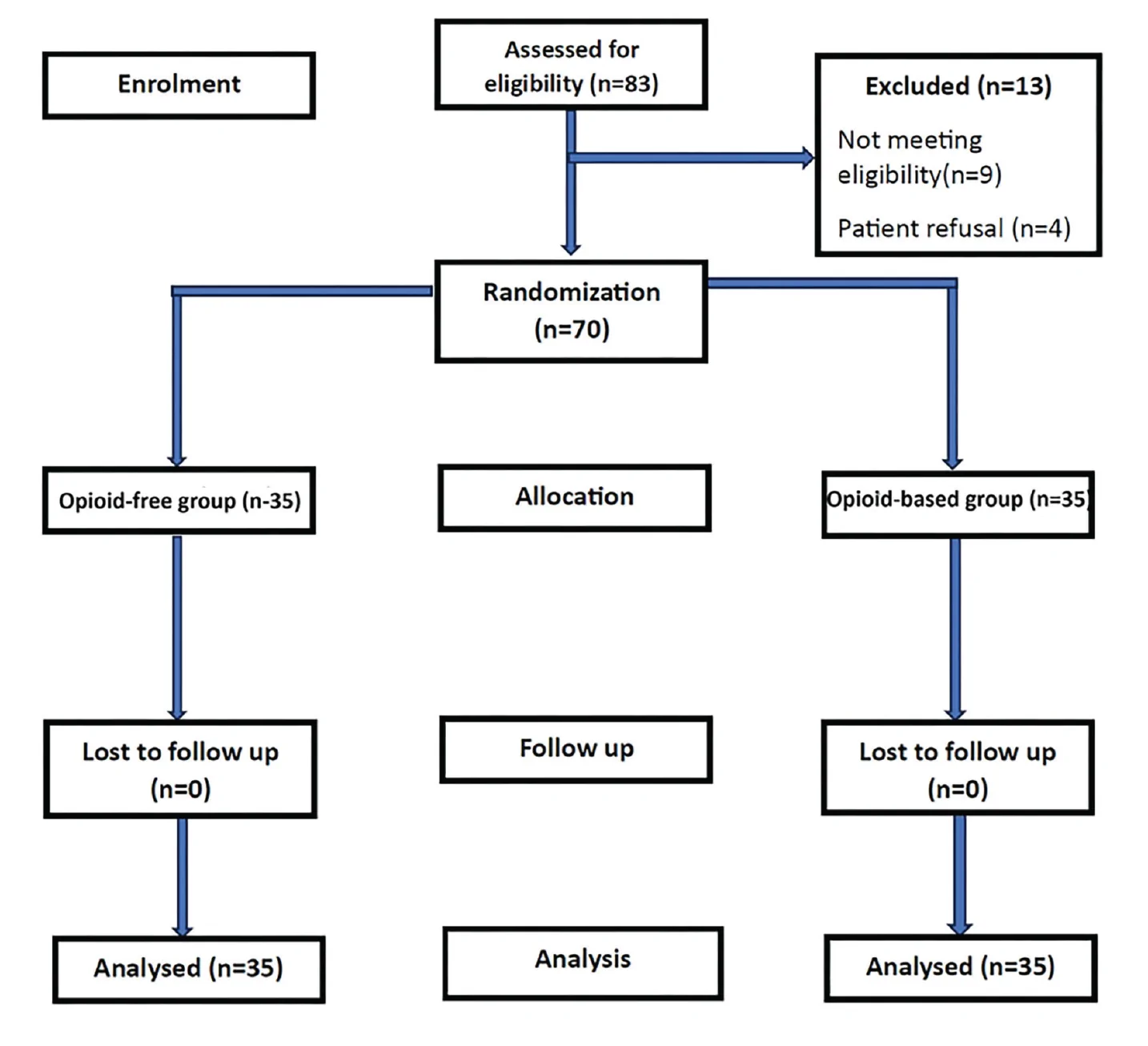
Consort flowchart.

**Figure 2:**
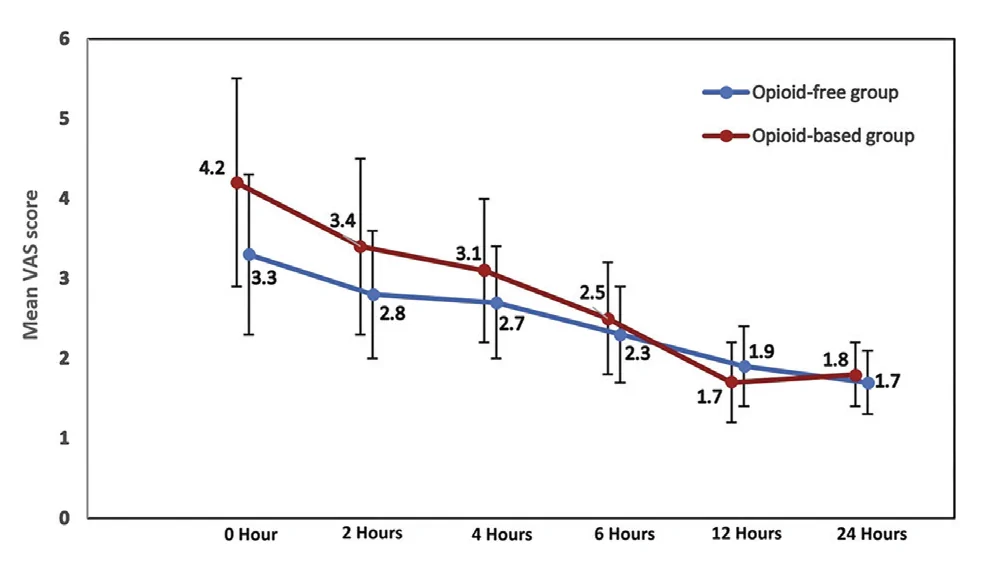
Postoperative mean VAS score at various time points among the 2 groups. Error bars represent standard deviation. VAS, visual analogue scale.

**Figure 3:**
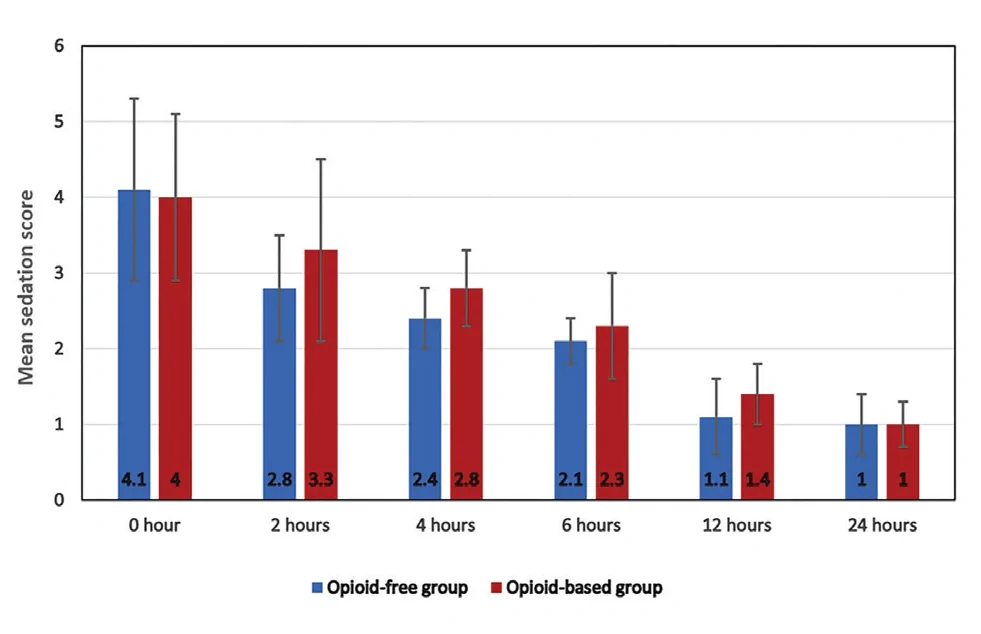
Mean Ramsay Sedation Scale at various time points among the 2 groups. Error bars represent standard deviation.

**Figure 4:**
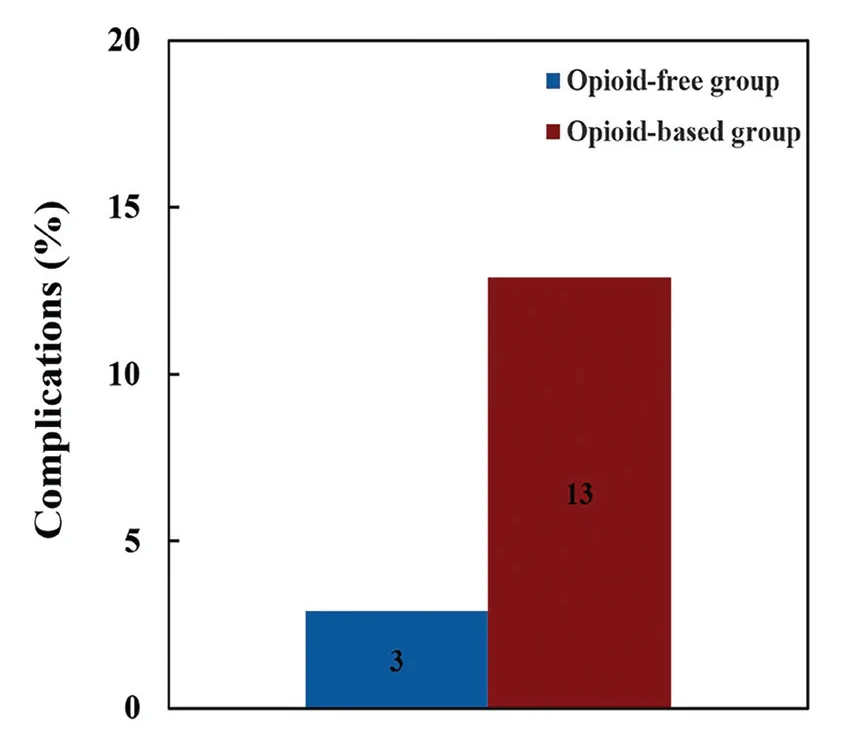
Distribution of overall complications among the 2 groups.

**Table 1. Comparison of Demographic and Clinical Characteristics Among the 2 Groups:** 

-	**Opioid-free group**	**Opioid-based group**	***P* value**
**Age (years), mean ± SD**	40.32±13.642	37.63±11.184	0.370
**Gender, n (%)**			0.454
Male	21 (60.0)	24 (68.6)	
Female	14 (40.0)	11 (31.4)	
**BMI (kg m^-2^), mean ± SD**	23.16±1.842	23.42±2.301	0.603
**ASA class, n (%)**			0.445
Class I	25 (71.4)	22 (62.9)	
Class II	10 (28.6)	13 (37.1)	
**Surgical procedure, n (%)**			0.337
Laparoscopic appendectomy	14 (40)	18 (51.4)	
Laparoscopic cholecystectomy	21 (60)	17 (48.6)	
**Duration of surgery, n (%)**			0.858
Less than 60 minutes	13 (37.2)	11 (31.4)	
60 to 120 minutes	18 (51.4)	19 (54.3)	
More than 120 minutes	4 (11.4)	5 (14.3)	

BMI, body mass index; ASA, American Society of Anesthesiologists physical status; SD, standard deviation

**Table 2. Distribution of Need for Rescue Analgesia and Time Taken for the First Dose of Rescue Analgesia Among the 2 Groups:** 

**Need for rescue analgesia**	**Opioid-free group n (%)**	**Opioid-based group n (%)**	**Total n (%)**	***P* value**
Yes	17 (48.6)	32 (91.4)	49 (70.0)	<0.001*
No	18 (51.4)	3 (8.6)	21 (30.0)	
Total	35 (100)	35 (100)	70 (100)	
**Time taken for the first dose of rescue analgesia**	**Opioid-free group** **n**	**Opioid-based group** **n**	**Total** **n**	***P* value**
Less than 6 hours	12	27	39	0.401
6-12 hours	4	3	7	
More than 12 hours	1	2	3	
Total	17	32	49	

*: Statistical significance

**Table 3. Comparison of Heart Rate Between the Two Groups at Different Intraoperative Time Points:** 

**Heart rate**	**Opioid-free group** **Mean ± SD**	**Opioid-based group** **Mean ± SD**	***P* value**
**Baseline**	86.1±4.8	87.5±5.2	0.245
**After** **intubation**	87.3±5.9	86.4±6.4	0.541
**Before** **CO_2_** **insufflation**	85.4±7.5	84.3±7.2	0.532
**10** **mins** **after** **CO_2_** **insufflation**	86.6±7.3	86.5±6.6	0.953
**30** **mins** **after** **CO_2_** **insufflation**	85.3±7.5	85.2±7.6	0.957
**60** **mins** **after** **CO_2_** **insufflation**	85.7±8.0	85.6±6.1	0.954
**After** **CO_2_** **deflation**	84.3±7.5	84.2±7.6	0.957
**After** **extubation**	82.7±8.3	83.6±6.5	0.614

CO_2_, carbon dioxide, SD, standard deviation

**Table 4. Comparison of Systolic Blood Pressure, Diastolic Blood Pressure, and Mean Arterial Pressure Between the Two Groups at Different Intraoperative Time Points:** 

**Time point**	**SBP opioid-free group** **Mean ± SD**	**SBP opioid-based group Mean ± SD**	**SBP** ***P* value**	**DBP opioid-free group** **Mean ± SD**	**DBP opioid-based group** **Mean ± SD**	**DBP** ***P* value**	**MAP opioid-free group** **Mean ± SD**	**MAP opioid-based group** **Mean ± SD**	**MAP** ***P* value**
**Baseline**	113.2±6.6	113.0±3.4	0.874	72.5±5.3	73.3±2.7	0.428	69.2±4.4	67.7±3.2	0.107
**After** **intubation**	114.8±5.1	114.3±9.1	0.778	71.8±6.9	72.0±1.4	0.867	68.4±5.4	66.3±3.9	0.067
**Before** **CO_2_** **insufflation**	112.3±6.7	112.1±3.7	0.878	73.9±4.1	72.3±3.9	0.098	68.3±5.8	66.6±3.5	0.165
**10** **mins** **after** **CO_2_** **insufflation**	115.7±8.4	111.3±7.8	0.025*	74.1±5.4	71.0±2.9	0.005*	70.3±5.4	67.4±3.4	0.012*
**30** **mins** **after** **CO_2_** **insufflation**	115.8±6.8	112.3±3.2	0.008*	75.2±4.2	72.1±2.1	<0.001*	69.3±5.1	67.2±2.9	0.037*
**60** **mins** **after** **CO_2_** **insufflation**	114.3±5.6	112.8±8.7	0.395	74.6±4.3	74.1±2.8	0.546	69.0±4.7	68.0±2.0	0.250
**After** **CO_2_** **deflation**	112.3±6.3	111.3±3.4	0.412	74.3±3.3	73.0±2.6	0.072	67.8±5.7	66.8±2.4	0.361
**After** **extubation**	114.3±6.2	114.5±2.3	0.859	74.1±5.4	74.0±3.4	0.926	68.3±5.5	67.5±2.0	0.409

*: Statistical significance

SBP, systolic blood pressure; DBP, diastolic blood pressure; MAP, mean arterial pressure; CO_2_, carbon dioxide

## References

[1] Ekstein P, Szold A, Sagie B, Werbin N, Klausner JM, Weinbroum AA (2006). Laparoscopic surgery may be associated with severe pain and high analgesia requirements in the immediate postoperative period.. Ann Surg.

[2] Benyamin R, Trescot AM, Datta S (2008). Opioid complications and side effects.. Pain Physician.

[3] Elvir-Lazo OL, White PF (2010). The role of multimodal analgesia in pain management after ambulatory surgery.. Curr Opin Anaesthesiol.

[4] Gürkan Y, Aksu C, Kuş A, Yörükoğlu UH, Kılıç CT (2018). Ultrasound guided erector spinae plane block reduces postoperative opioid consumption following breast surgery: a randomized controlled study.. J Clin Anesth.

[5] Ragupathy R, Prabhu SCG, Thiyagarajan D, Anto V (2022). Opioid-free anaesthesia for laparoscopic surgeries - a prospective non-randomised study in a tertiary care hospital.. Indian J Anaesth.

[6] Bohringer C, Astorga C, Liu H (2020). The benefits of opioid free anesthesia and the precautions necessary when employing it.. Transl Perioper Pain Med.

[7] Dunn LK, Durieux ME (2017). Perioperative use of intravenous lidocaine.. Anesthesiology.

[8] Gulabani M, Gurha P, Dass P, Kulshreshtha N (2015). Comparative analysis of efficacy of lignocaine 1.5 mg/kg and two different doses of dexmedetomidine (0.5 μg/kg and 1 μg/kg) in attenuating the hemodynamic pressure response to laryngoscopy and intubation.. Anesth Essays Res.

[9] Lee IW, Schraag S (2022). The use of intravenous lidocaine in perioperative medicine: anaesthetic, analgesic and immune-modulatory aspects.. J Clin Med.

[10] Shin HJ, Na HS, Do SH (2020). Magnesium and pain.. Nutrients.

[11] Forero M, Adhikary SD, Lopez H, Tsui C, Chin KJ (2016). The erector spinae plane block: a novel analgesic technique in thoracic neuropathic pain.. Reg Anesth Pain Med.

[12] Elshafie MA, Khalil MK, ElSheikh ML, Mowafy NI (2022). Erector spinae block with opioid free anesthesia in cirrhotic patients undergoing hepatic resection: a randomized controlled trial.. Local Reg Anesth.

[13] Koo CH, Hwang JY, Shin HJ, Ryu JH (2020). The effects of erector spinae plane block in terms of postoperative analgesia in patients undergoing laparoscopic cholecystectomy: a meta-analysis of randomized controlled trials.. J Clin Med.

[14] Aronsohn J, Zeltsman M, Gerasimov M, Palleschi G, Yang P, Dowling O (2021). Opioid-free anesthesia in bariatric surgery: a retrospective cohort study.. Int J Anesthetic Anesthesiol.

[15] Choi H, Song JY, Oh EJ, Chae MS, Yu S, Moon YE (2022). The effect of opioid-free anesthesia on the quality of recovery after gynecological laparoscopy: a prospective randomized controlled trial.. J Pain Res.

[16] Massoth C, Schwellenbach J, Saadat-Gilani K (2021). Impact of opioid-free anaesthesia on postoperative nausea, vomiting and pain after gynaecological laparoscopy - a randomised controlled trial.. J Clin Anesth.

[17] Toleska M, Dimitrovski A (2019). Is opioid-free general anesthesia more superior for postoperative pain versus opioid general anesthesia in laparoscopic cholecystectomy?. Pril (Makedon Akad Nauk Umet Odd Med Nauki).

